# Interspecies Haptic Sociality: An Observation of Grooming Between Two Mongoose Species

**DOI:** 10.1002/ece3.71659

**Published:** 2025-06-21

**Authors:** Kyle Smith, Malcolm Hepplewhite, Emmanuel Do Linh San, Michael J. Somers

**Affiliations:** ^1^ Mammal Research Institute, Department of Zoology and Entomology University of Pretoria Pretoria South Africa; ^2^ Independent Researcher Pretoria South Africa; ^3^ Department of Biological and Agricultural Sciences Sol Plaatje University Kimberley South Africa; ^4^ Centre for Invasion Biology, Department of Zoology and Entomology University of Pretoria Pretoria South Africa

**Keywords:** *Cynictis penicillata*, interspecific interaction, mutualism, *Suricata suricatta*, suricate, yellow mongoose

## Abstract

Meerkats ( *Suricata suricatta* ) and yellow mongooses ( *Cynictis penicillata* ) share many behavioural characteristics and are known to, on rare occasions, live in close association through displayed cooperative vigilance and shared burrow use. Here, we describe the first visual observation of tactile social behaviour through grooming between a meerkat and a yellow mongoose in the Rietvlei Nature Reserve, South Africa. We hypothesise that the close relationship between the two species in the reserve may be a response to a combination of phylogenetic ties, shared behavioural traits, and the population collapse of meerkats in the reserve that exposed a vacant social niche. This observation of interspecific sociality further extends our knowledge of cooperation and group augmentation among meerkats, yellow mongooses and carnivores in general.

## Graphical abstract

This study presents the first visual observation of grooming between a meerkat ( *Suricata suricatta* ) and a yellow mongoose ( *Cynictis penicillata* ) in the Rietvlei Nature Reserve, South Africa. We hypothesise that this rare interspecific social behaviour may be a result of phylogenetic ties, shared behaviours and a recent collapse of the resident meerkat population. This observation expands our understanding of cooperation and social behaviour among these species and carnivores in general.

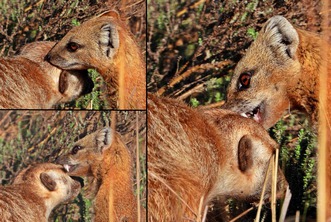

## 1 Introduction

Yellow mongooses ( *Cynictis penicillata* ) and meerkats ( *Suricata suricatta* ) are small carnivores of the Herpestidae family. Apart from differences in sociality between the two species, they share many behavioural traits, such as being active during the same periods of the day, co‐existing in open environments, having similar diets and facing similar predatory threats (Lynch 1980). Meerkats are obligate social mongooses with complex group structures and social dynamics (Schneider and Kappeler 2014). Yellow mongooses, however, are facultatively social, solitary foragers, meaning that although they usually forage alone, they form gregarious groups of between two and 14 individuals that usually meet up at dens (Balmforth 2004; Cavallini 1993; le Roux 2007; Rasa et al. 1992). Co‐existence between the two species extends beyond living in sympatry; communal home burrow systems are sometimes shared (Do Linh San and Somers 2006; Howard 1994; le Roux et al. 2009), and cooperative vigilance has been observed (Do Linh San and Somers 2006). Here, we describe an observation of grooming between a yellow mongoose and a meerkat that further extends our understanding of interspecific sociality among yellow mongooses and meerkats and carnivores in general.

## 2 Field Observation

**FIGURE 1:**
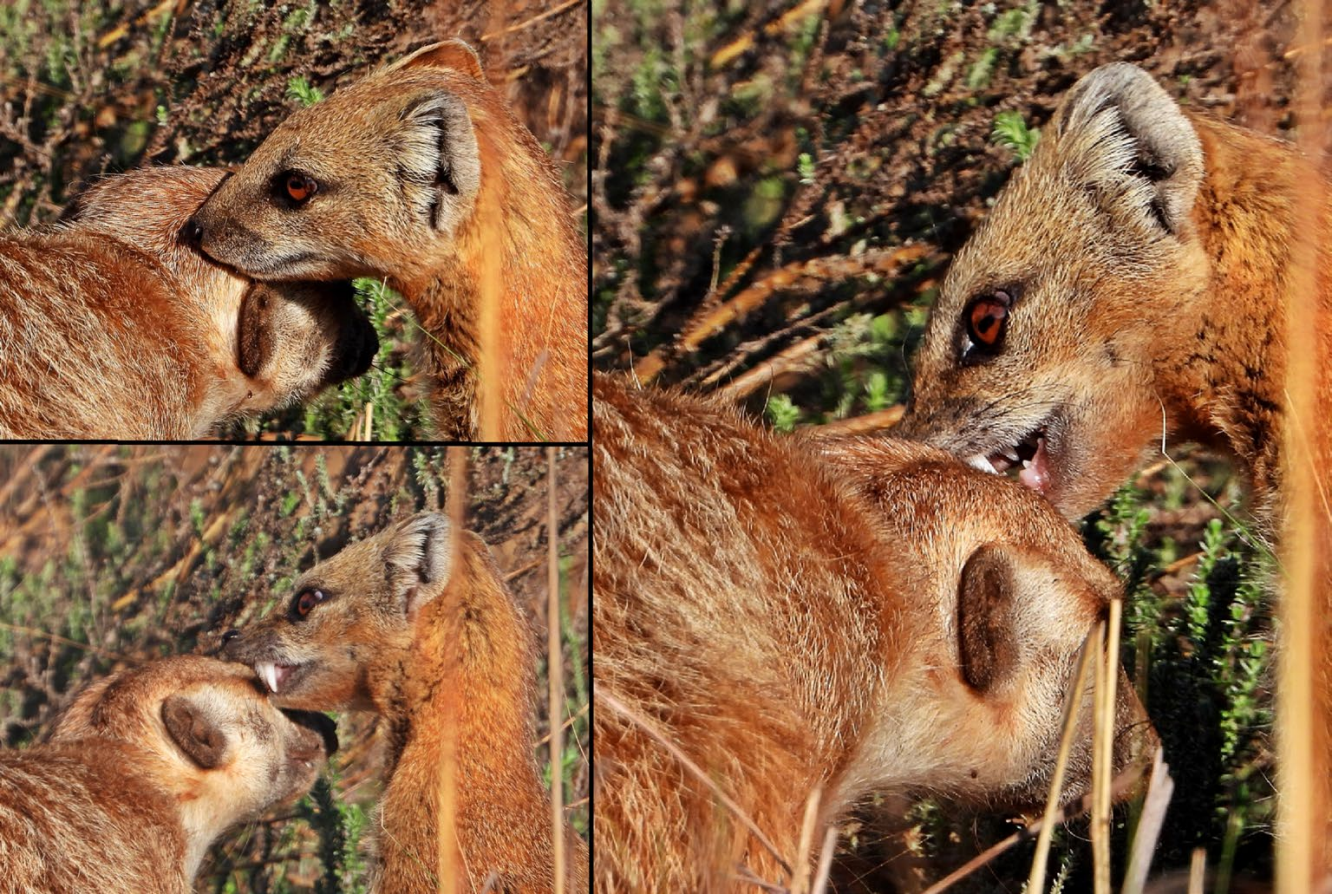
Photographs taken by MH on the 25 October 2024 of a yellow mongoose ( *Cynictis penicillata* ) (right) grooming a meerkat ( *Suricata suricatta* ) (left) in the southern section of the Rietvlei Nature Reserve, South Africa. The meerkat was also observed grooming the yellow mongoose, but this behaviour was not recorded. The interaction lasted for approximately 4 min.

The visual observation occurred in the Rietvlei Nature Reserve (RNR), located near the city of Pretoria in the Gauteng province, South Africa, at 25.8825° S, 28.2639° E. The 40 km^2^ reserve is located in the Rand Highveld Grassland region of the country's Grassland Biome, meaning that the environment is predominantly made up of open grasslands with few isolated patches of woodland (Mucina and Rutherford 2006). The observation was made, and the behaviours were photographed by MH on the 25 October 2024 from a vehicle while in the southern section of the reserve. The observer followed two meerkats moving along the verge of the road for approximately 500 m when the two individuals separated. One individual met up with a yellow mongoose close to the road when the social interaction commenced. The yellow mongoose was seen grooming the meerkat in a nibbling manner (Figure 1). The meerkat reciprocated in grooming the yellow mongoose, but for a much shorter period. No aggression was observed between the two species. The amicable interaction lasted for approximately 4 min before the two individuals separated and moved away from each other.

## 3 Discussion

Heterospecific tolerance and mutualism are not new phenomena between meerkats and yellow mongooses. Yet, current knowledge only describes mutualistic benefits of cooperation in terms of antipredator behaviour (Do Linh San and Somers 2006) and has not included haptic sociality (positive tactile communication or information gained from other individuals through touch), where grooming affirms relationships and perhaps strengthens bonds between individuals of the two species. Cooperative vigilance and shared burrow use align with adaptive behaviour in response to an exposed environment with high predation pressure (Howard 1994; Rasa 1987). Many diurnal herpestid species that inhabit open environments, such as meerkats, dwarf mongooses ( *Helogale parvula* ) and banded mongooses ( *Mungos mungo* ), have evolved a high degree of sociality (Rasa 1987; Rood 1987; Schneider and Kappeler 2014). Apart from increased mating opportunities and access to information that is likely by‐products of social group formation, the high degree of sociality in small diurnal insectivorous herpestid species might rather be explained by the ‘safety in numbers’ approach where group defence based on a symbiotic relationship proved to be a more effective anti‐predator mechanism than solitary living (Rasa 1987; Rood 1987; Schneider and Kappeler 2014). The high degree of cooperation described is not limited to intraspecific boundaries, for positive interactions between species and the formation of mixed‐species groups that benefit from shared social behaviour are known to occur across taxa (Goodale et al. 2020; Makenbach et al. 2013; Oliveira and Bshary 2021; Stensland et al. 2003). In mixed‐species groups of large and medium‐sized mammalian herbivores, migratory species such as blue wildebeest ( *Connochaetes taurinus* ) and common zebra ( *Equus quagga* ) form groups in areas of high forage quality (Saltz et al. 2023), and multi‐species groups of ungulates benefit from more effective predator vigilance and improved foraging time (Creel et al. 2014; Fitzgibbon 1990; Morse 1977). Mutualism through cooperative vigilance also exists between certain groups of dwarf mongooses and hornbills (Rasa 1983). In the current context, interspecies grooming has been observed several times among primates (Freymann et al. 2023; Lee et al. 2021), and certain bird species, such as oxpeckers (*Buphagus* spp.), which commonly groom mammals (Nunn et al. 2011).

Specifically concerning the relationship between meerkats and yellow mongooses, a high degree of interspecific tolerance seen through shared burrow use, social behaviour, group foraging and cooperative vigilance is likely a proactive response associated with the benefits of group augmentation (forming larger social groups to benefit survival and fitness) that is primarily driven by predator avoidance (Creel et al. 2014; Do Linh San and Somers 2006; Howard 1994). In RNR, an acute and significant decrease in meerkat numbers occurred in 2023, where it is estimated by reserve staff that only one or two individuals remain (Tshwane Conservation Personal Communication, 13 March 2025). The cause of the population decrease is unknown, but possible reasons may include disease, permanent movement out of the reserve or increased predation.

The observation of grooming between a yellow mongoose and a meerkat described here confirms a strong interspecific relationship. Allogrooming (the act of grooming another individual of the same species) is rarely observed among yellow mongooses (Cavallini 1993; Howard 1994) but is common among meerkats, where dominant individuals will be groomed more often and for longer periods than subordinates (Kutsukake and Clutton‐Brock 2006, 2010). It may be speculated that the yellow mongoose in the observation above was subordinate to the meerkat and may have attempted to placate the meerkat to avoid antagonism or to merely maintain the social bond between the pair, mutually benefitting the survival and fitness of both (Kutsukake and Clutton‐Brock 2006, 2010). The asymmetrical grooming efforts observed further support the idea that the meerkat is dominant in the interaction (Kutsukake and Clutton‐Brock 2006, 2010). A possible abrupt collapse of the RNR meerkat population may have triggered a relaxation of social tolerance between the meerkat and yellow mongoose, which the former social species allowed due to small group size and the latter, less social mongoose, perhaps capitalised on to improve predation avoidance through social group augmentation and its complementary advantages of cooperative vigilance; an adaptive social strategy built from a reactive response for a proactive approach where benefits likely outweigh costs of close association (Creel et al. 2014). In addition, yellow mongoose group sizes increase during the breeding season (le Roux et al. 2008), mainly during the wet summer months from October to February (Rasa et al. 1992). Although unconfirmed for the region, this may coincide with the onset of the peak breeding season of meerkats (Doolan and Macdonald 1997; Thorley et al. 2025). The influence of behavioural changes, especially by the yellow mongoose during their breeding season, could induce an increased benefit of a mutualistic relationship between the two species during this period (Howard 1994). The month when the current observation of interspecies grooming was made (October) falls within this potential period when behavioural changes are likely to occur.

In conclusion, the described observation of grooming between a yellow mongoose and a meerkat is a rare case of interspecific haptic sociality based on a foundation of group augmentation that is likely in response to a combination of phylogenetic ties, shared behavioural traits, and the population collapse of meerkats in RNR that exposed a vacant social niche. Further research is, however, required to confirm the cause of the close association between the two mongoose species in RNR. This could improve our understanding of the evolution of sociality in small carnivores.

## Author Contributions

**Kyle Smith:** conceptualization (equal), investigation (lead), writing – original draft (lead), writing – review and editing (equal). **Malcolm Hepplewhite:** data curation (lead), writing – review and editing (equal). **Emmanuel Do Linh San:** conceptualization (equal), writing – original draft (supporting), writing – review and editing (equal). **Michael J. Somers:** conceptualization (equal), writing – original draft (supporting), writing – review and editing (equal).

## Conflicts of Interest

The authors declare no conflicts of interest.

## Data Availability

Data sharing is not applicable to this article as no data sets were generated or analysed during the current study.
